# Mitochondrial metabolic-transcriptional axis drives adaptive drug tolerance across major lung cancer subtypes

**DOI:** 10.3389/fgene.2026.1958851

**Published:** 2026-09-22

**Authors:** Ruixuan Liu, Xinyao Wang, Zetong Li, Weijie Mao, Wenjing Zhang, Chunchen Wang, Zhuoran Tang

**Affiliations:** 1 Department of Pathology, Medical School of Nantong University, Nantong, China; 2 Department of Respiratory, Affiliated Huishan Hospital of Xinglin College, Nantong University, Wuxi Huishan District People’s Hospital, Wuxi, China

**Keywords:** drug resistance, drug-tolerant persister cells, epigenetic regulation, lung cancer, metabolic reprogramming, mitochondrial metabolism, transcriptional resilience

## Abstract

Drug resistance remains the first and most significant barrier to long-term curative benefits in lung cancer patients. Traditional resistance mechanisms focused on oncogenic driver mutations are inadequate to explain reversible tumor phenotypic plasticity under therapeutic stress. Increasing evidence has placed mitochondrial metabolic rewiring as the core signaling hub bridging energy homeostasis to nuclear epigenetic reprogramming; however, a comparative review of this metabolic-transcriptional axis in lung adenocarcinoma (LUAD), lung squamous cell carcinoma (LUSC), and small cell lung cancer (SCLC) remains underrepresented. Here, this review integrates the published preclinical and translational data, dissecting how mitochondrial dysfunction, including altered oxidative phosphorylation (OXPHOS), TCA metabolite accumulation, ROS imbalance, and defective mitochondrial quality control, establishesdrug-tolerant persister (DTP) states. Subsequently, we elaborate on the conserved and subtype-specific genetic and epigenetic cascades induced by mtDNA variants, oncogenes (MYC/RAS), tumor suppressors (p53/LKB1), and oncometabolites (α-ketoglutarate, succinate, fumarate) that remodel chromatin landscapes and further initiate lineage plasticity, epithelial-mesenchymal transition, and immune evasion. We summarize promising mitochondrial-targeted interventions from OXPHOS inhibitors, fatty acid oxidation blockers, ROS modulators, and epigenetic agents and propose combinatorial therapeutic regimens according to the three lung cancer subtypes. This work establishes a unified metabolic-resilience framework and highlights multi-omics integrated profiling as a new paradigm in precision medicine to forecast resistance risk and reverse therapeutic failure in lung cancer.

## 1 Introduction: the role of mitochondria in lung cancer progression and therapy resistance

### 1.1 Overview of lung cancer subtypes and current treatment challenges

Lung cancer is one of the most common malignant tumors and the leading cause of cancer death worldwide. Histopathologically, lung cancer is subdivided into non-small cell lung cancer (NSCLC, predominantly lung adenocarcinoma (LUAD) and lung squamous cell carcinoma (LUSC)) and small cell lung cancer (SCLC), with marked intersubtype divergence in biological properties, molecular profiles and therapeutic responsiveness. In recent years, the emergence of targeted therapy and immune checkpoint inhibitors has revolutionized the field of oncology (Du et al., 2025b). Drugs targeting driver genes such as EGFR, ALK and KRAS have brought significant survival benefits to some patients (Zhou et al., 2025). However, although targeted therapy, chemotherapy and immunotherapy have improved the survival rate of patients, the most serious challenge in the treatment of lung cancer is drug resistance, especially acquired drug resistance (especially DTP cell-mediated drug resistance) (Liu et al., 2025). The development of drug resistance leads to tumor progression and shorts the window of long-term benefit of previous treatment strategies.

### 1.2 Mitochondrial metabolism and adaptation of cancer cells

Cancer metabolism research has long been dominated by the “Warburg effect”, in which cancer cells meet their energy demands for rapid growth and proliferation by turning on aerobic glycolysis. However, the latest evidence shows that the metabolism of cancer cells is not an unchanging ‘Warburg phenotype’, but a highly dynamic adaptive process. Lung-cancer cells exhibit an impressive metabolic plasticity to dynamically reprogram the tricarboxylic acid (TCA) cycle, fatty acid oxidation, and glutamine metabolism and to switch to oxidative phosphorylation (OXPHOS) under therapeutic stress (including chemotherapy or targeted therapy) to sustain energy homeostasis and biosynthetic demand (Lin et al., 2025). This metabolic reprogramming is not only a change in the energy supply but also a profound reshaping of the cellular biochemical environment: by altering the levels of crucial metabolic intermediates such as α-ketoglutarate, succinate and fumarate, mitochondria directly influence the activity of DNA and histone modifying enzymes, thereby driving the remodeling of the epigenetic landscape (Sun et al., 2021). Moreover, as important signaling molecules, ROS may activate different kinds of transcription factors and stress pathways, which also promote the expression of anti-apoptosis and DNA damage repair genes, allowing cancer cells to survive and proliferate in the therapeutic stress environment (Luis et al., 2021). This “metabolics-epigenetics-transcription” cascade constitutes the core mechanism for the survival and evolution of cancer cells under therapeutic stress, and this metabolites-mediated epigenetic reprogramming is a key breakthrough point for our understanding of tumor drug resistance and heterogeneity.

### 1.3 Mitochondrial metabolic reprogramming: key mechanisms driving transcriptional and epigenetic remodeling and leading to drug resistance in lung cancer

Although driver gene mutation and bypass signal activation are the classical mechanisms of drug resistance in lung cancer, they are difficult to fully explain the highly reversible phenotypic plasticity shown by tumor cells under therapeutic pressure (Du et al., 2025a; Huang et al., 2025). Accumulating evidence suggests that the essence of this adaptive survival lies in the remodeling of the “metabolism-transcriptional axis”: that is, the mitochondrial metabolic state acts as a dynamic signaling hub to directly regulate the gene expression profile in the nucleus, thereby conferring drug resistance to cancer cells.

The aim of this review is to systematically elaborate how the metabolic-transcriptional axis mediates drug resistance separately in lung adenocarcinoma (LUAD), lung squamous cell carcinoma (SCC) and small cell lung cancer (SCLC), the three major pathological subtypes of lung cancer defined above. In this review, we define the metabolic-transcriptional axis as a multi-layered signaling cascade with feedback loops that couples mitochondrial metabolic output to nuclear gene expression. The axis comprises four functionally interconnected layers: Metabolic Input, Enzymatic Transducers, Chromatin Output, Transcriptional Response. Critically, the MTA is not a linear pathway but a self-reinforcing circuit. For example, MYC-driven mitochondrial biogenesis and NRF2-mediated antioxidant defense feed back to Layer 1, while epigenetic silencing of metabolic checkpoints (e.g., LKB1) further amplifies rewiring. This positive feedback architecture explains how the initially reversible DTP state progressively stabilizes into durable resistance.

## 2 Mitochondrial metabolic alterations in lung cancer

Mitochondrial metabolic reprogramming in lung cancer encompasses multiple core metabolic processes, including oxidative phosphorylation (OXPHOS), the tricarboxylic acid (TCA) cycle, and reactive oxygen species (ROS) homeostasis. This metabolic rewiring endows tumor cells with superior survival advantages and is tightly implicated in the emergence of therapeutic drug resistance.

Studies related to cancer cells have shown a simultaneous increase in both OXPHOS and glycolysis levels in tumor tissues (Gao et al., 2016; Yu et al., 2017). Increased OXPHOS activity maintains a stable ATP production and is highly correlated with the establishment of drug-resistant tumor cells (see below). Recent evidence shows upregulation of electron transport chain and OXPHOS related genes like NDUF, UQCR and ATP5D in lung adenocarcinoma. These molecular alterations boost mitochondrial respiration, providing tumor cells with sufficient ATP and critical metabolic intermediates to support malignant growth (Kalainayakan et al., 2018). In the H446 small cell lung cancer cell line, subsets of cancer stem cells (CSCs) are more sensitive to OXPHOS inhibitors (e.g., oligomycin) than non-stem cell subsets. The result suggests that cancer stem cells are more dependent on OXPHOS for energy supplementation even though the overall oxygen consumption rate (OCR) is lower (Gao et al., 2016). However, metabolic phenotype characterized by enhanced glycolysis and inhibited OXPHOS is observed in some squamous cell carcinoma samples in NSCLC mouse models and human cell lines (Momcilovic et al., 2019). Taken together, these lines of evidence suggest metabolic heterogeneity in tumor lesions.

In tumors, TCA cycle performs two essential biological functions, such as maintaining energy metabolism and generating abundant signaling-active metabolites. These molecules influence cell fate, remodel the tumor immune niche and modify cellular drug sensitivity through epigenetic reprogramming and intracellular signaling cascades (Todisco et al., 2019). Significant heterogeneity of the TCA cycle exists in multiple lung cancer models with different genetic backgrounds that underlie subtype-specific drug resistance (Davidson et al., 2016). Furthermore, metaboloproteomic analysis of 128 lung adenocarcinoma samples shows significant heterogeneity in expression of mitochondrial enzymes involved in the TCA cycle, fatty acid oxidation and OXPHOS. This heterogeneous molecular signature is strongly associated with patient overall survival and risk of postoperative relapse (Torresano et al., 2022). So far fumarate has got the most sufficient experimental evidence in lung cancer research. Functional experiments have confirmed that fumarate accumulation can inhibit TET2 activity, disrupt DNA methylation and promote epithelial-mesenchymal transition (EMT), tumor cell migration, invasion and *in vivo* metastasis (Ding et al., 2026). Studies of the accumulation of other TCA metabolites are mainly focused on other tumor types at present.

Electrons “leak” from the respiratory chain in mitochondria to generate superoxide, H2O2 and other ROS. Excessive reactive species damage proteins, lipids and genomic DNA, ultimately leading to cell death (Holze et al., 2018). The ROS production has been shown in the data to be at a high level in lung cancer cells, particularly in the drug-resistant populations. High levels of ROS are to promote “adaptive oxidative stress” and not to constitute an immediate lethal threshold, high ROS levels maintain “adaptive oxidative stress.” For example, compared with parental cells, EGFR-TKI-resistant PC9 T20 and HCC827 T20 cells in NSCLC have higher mtOXPHOS activity and ROS levels. This state does not induce cell death but allows the generation of CSC-like subpopulations with significant survival advantages (Li et al., 2015). Tumor cells also activate antioxidant cascades under adaptive oxidative stress to enhance survival fitness. In zebrafish-based NSCLC models, ROS elevation induced by hydrogen peroxide favors PRDX5–NRF2 complex assembly. The close relationship between the functional linkage of two proteins and the accelerated proliferation of NSCLC and acquired drug resistance suggests that the NRF2-controlled antioxidant machinery enables malignant cells to survive in a high ROS environment (QIAN et al., 2022). Furthermore, chronic intermittent hypoxia (CIH) causes mtROS accumulation in mouse lung cancer models. Mitochondrial ROS induces Bach1 expression which upregulates CSC marker expression and CSC proportion, leading to enhanced tumor proliferation and metastasis (Hao et al., 2021).

Furthermore, pan-cancer studies have shown that remodeling mitochondrial morphology and quality control systems is a common feature in tumors, thus enhancing cellular tolerance to therapeutic stress (Rodrigues and Ferraz, 2020). Classic NSCLC studies have reported DRP1 upregulation and MFN2 downregulation, resulting in severe mitochondrial fragmentation (Rehman et al., 2012). Conversely, several acquired drug-resistant tumor models, including paclitaxel-resistant A549/Taxol and cisplatin-resistant SKOV3/DDP cells, exhibit enhanced mitochondrial fusion that strengthens tumor drug resistance (Zhou et al., 2020). Collectively, these findings suggest that both excessive mitochondrial fission and intensified fusion reflect an active disruption of the intrinsic fission-fusion balance, allowing cancer cells to evade apoptotic elimination and metabolic crisis. Furthermore, mitochondrial autophagy is closely implicated in tumor drug resistance. RFX2–BNIP3 axis-triggered mitochondrial autophagy acts as a key mechanism driving adaptive tolerance to ACK1-targeted therapy in NSCLC, and combined blockade of mitochondrial autophagy can markedly improve the efficacy of targeted treatment (Cao et al., 2025). In lung adenocarcinoma, the PINK1–Parkin axis activates mitochondrial autophagy to sustain mitochondrial function and redox homeostasis in drug-tolerant persister (DTP) cells, thereby mediating metabolic reprogramming and drug resistance upon MAPK pathway inhibition (Li et al., 2023). The PINK1/Parkin and BNIP3/NIX pathways are core modules for tumor mitochondrial autophagy and quality control, which closely link chemotherapy, targeted therapy, and radiotherapy resistance. Coordinated mitochondrial dynamics and autophagy ensure cancer cells have sufficient high-quality, functional mitochondria to maintain steady OXPHOS, ATP production, and antioxidant defense capacity (Table 1).

**TABLE 1:** Comparative analysis of OXPHOS-dependent metabolic enzyme Mutations,and drug resistance-associated transcription factors across three lung cancer subtypes.

Comparison dimension	Lung adenocarcinoma (LUAD)	Lung squamous cell carcinoma (LUSC)	Small cell lung cancer (SCLC)
Dominant Metabolic Phenotype	IDH1/2 Mutations: Frequency of approximately 0.8%–1%; drives 2-HG accumulation, inhibits TET/KDM6A, leading to CIMP phenotype and elevated H3K27me3 (Zhang et al., 2023). FH Downregulation: Causes fumarate accumulation, inhibits TET2, silences miR-200 family, and promotes EMT (Vadhan et al., 2023). mtDNA POLG Mutations: Increases mutational burden, forcing cells to rely on serine biosynthesis and glycolytic compensation (Cararo Lopes et al., 2025)	SDHA/SDHAF2 Mutations: Leads to succinate accumulation, inhibits TET/KDM6A and PHDs, stabilizes HIF-1α, and promotes glycolysis and anti-apoptosis (Kuo et al., 2022). FH Downregulation: Fumarate accumulation mediates epigenetic remodeling and metastasis (Vadhan et al., 2023). Metabolic Phenotype: Some samples exhibit a high-glycolysis/low-OXPHOS phenotype, whereas drug-resistant strains are often accompanied by enhanced mitochondrial fusion mediated by the p32/OPA1 axis (Yu et al., 2024)	mtDNA MT-CO1 Variants: Identified in PI3K/mTOR inhibitor-resistant models (e.g., H1975), driving metabolic reprogramming and excessive glycolysis (Koh et al., 2017). Mitochondrial Respiratory Chain Gene Expression: Electron transport chain genes such as NDUF, UQCR, and ATP5D are highly expressed in CSC subpopulations, maintaining high OXPHOS dependence (Gao et al., 2016). IDH/SDH/FH Mutations: High-frequency mutations in SCLC are not explicitly mentioned in the text; the focus is primarily on mtDNA mutations and respiratory chain functional dependence
Drug Resistance-Associated Transcription Factors and Key Transcriptional Regulators	NRF2: Regulated by the DPP4-MEK axis, enhancing antioxidant defense, maintaining mitochondrial activity, and mediating EGFR-TKI tolerance (ZHANG et al., 2025b). SP1/ERRα: In EGFR-TKI resistance, cholesterol accumulation activates EGFR/Src/Erk, inducing SP1 nuclear translocation and ERRα re-expression to regulate ROS detoxification (Pan et al.). HIF-1α: Regulated by SIRT3 deacetylation; SIRT3 inactivation leads to HIF-1α accumulation, promoting glycolysis and chemoresistance (Zhao et al.). FOXK2: ROS-OGT axis-mediated O-GlcNAcylation promotes nuclear translocation and activates SLC7A11, inhibiting ferroptosis (Zhang et al.).	NRF2: Assembly of the PRDX5-NRF2 complex mediates adaptive oxidative stress and acquired resistance (Qian et al., 2022). Bach1: Mitochondrial ROS induces Bach1 upregulation, increasing CSC marker expression and promoting proliferation and metastasis (Hao et al., 2021). FOXO3a: Activates PINK1/Parkin-mediated protective mitophagy, leading to radiotherapy resistance (Zhang et al., 2024). HIF-1α: Regulated by the SIRT1/PGC-1α/PAR-γ axis to control mitochondrial biogenesis, inducing chemoresistance (Xu et al., 2021)	NRF2: Maintains survival advantage in CSC subpopulations through antioxidant cascades (Li et al., 2015). HIF-1α: Stabilized under hypoxia and metabolic stress, synergizing with the high proliferation and drug resistance characteristics of SCLC (Kuo et al., 2022). ABC Transporter Regulation: Transcriptional regulation of ABCB1/ABCG2 overexpression mediates multidrug resistance to platinum agents, topoisomerase inhibitors, and TKIs (Robey et al., 2018)

## 3 Genetic and molecular mechanisms linking mitochondrial metabolism to drug resistance

### 3.1 Mitochondrial DNA (mtDNA) mutation and compensatory metabolic reprogramming

In lung cancer, mtDNA mutations frequently impair mitochondrial respiratory function. Tumor cells develop adaptive metabolic compensation to sustain viability, and such compensatory rewiring is tightly associated with chemoresistance and targeted therapy resistance. Distinct mtDNA status exerts divergent impacts on tumor proliferation. Mutations in mitochondrial DNA polymerase γ increase the mtDNA mutational burden in non-small cell lung cancer (NSCLC) and compromise mitochondrial function. To compensate, tumor cells upregulate glycolysis and become highly glucose-dependent, accompanied by a declin (Cararo Lopes et al., 2025) ed NAD^+^/NADH ratio and suppressed *de novo* serine synthesis. As serine is indispensable for anabolic metabolism and redox homeostasis, NSCLC cells reserve functional mtDNA to sustain serine production and proliferation; accordingly, pharmacological blockade of serine synthesis abolishes metabolic compensation and restrains tumor growth.

Acquired resistance to PI3K/mTOR inhibitors in H1975 cells is accompanied by MT-CO1 variant in mtDNA; such genomic alteration drives metabolic reprogramming and excessive glycolysis, verifying that mtDNA aberration-initiated metabolic remodeling is an intrinsic driver of targeted drug resistance (Koh et al., 2017).

Cancer cells can transfer mutated mitochondria to tumour-infiltrating lymphocytes (TILs) within the tumour microenvironment. These mitochondria, having been transferred into TILs, can evade the mitophagic clearance process within TILs by binding to autophagy-inhibitory molecules, thereby causing homozygous replacement of the TILs’ endogenous mtDNA. This ultimately leads to metabolic dysfunction and cellular senescence in the lymphocytes, significantly weakening the body’s anti-tumour T-cell immunity and ultimately facilitating tumour immune escape. This mitochondrial intercellular trafficking between malignant cells and immune cells represents a novel metabolic-epigenetic regulatory mechanism governing tumour immunity (Ikeda et al., 2025). Furthermore, intracellular mtDNA depletion or mtDNA mutations can attenuate mitochondrial apoptotic signalling by reducing ROS production and inhibiting the full activation of Bax/caspases, ultimately leading to cisplatin resistance in tumour cells. Targeted intervention in mtDNA using platinum-mtDNA conjugates, however, can enhance mitochondrial-mediated apoptosis and effectively reverse drug insensitivity in tumour cells (van Gisbergen et al., 2015).

### 3.2 Altered expression of metabolic enzymes and transporters in drug-resistant cells

In non-small cell lung cancer (NSCLC), the expression profile of metabolic enzymes and transporters undergoes significant remodelling; this change directly regulates mitochondrial function and constitutes an important molecular basis for the development of drug resistance in tumours.

In drug-resistant cells, key metabolic enzymes are upregulated, thereby enhancing oxidative phosphorylation (OXPHOS) to maintain cellular energy supply and anti-apoptotic capacity. For example, the binding of TIM4 to ANXA2 activates PI3K-AKT signalling, which in turn upregulates the mitochondrial fusion protein L-OPA1, ultimately increasing the cellular oxygen consumption rate, membrane potential and the number of healthy mitochondria under normal conditions (Wang et al., 2023). In a cisplatin-resistant NSCLC model, elevated OPA1 expression promotes mitochondrial hyperfusion, ATP production, and resistance to cisplatin-induced apoptosis (Yu et al., 2024). It can also induce DPP4, yet activate the MEK-NRF2 signaling pathway, leading to the upregulation of antioxidant pathways to keep the mitochondria working and the level of ROS low while promoting fatty acid oxidation through CPT1A to shift cellular metabolism from glycolysis to OXPHOS (ZHANG et al., 2025a). Several reviews suggest this, including that most drug-resistant tumor cells, metastatic tumor cells, and cancer stem cells rely on mitochondrial OXPHOS; thus, the activated OXPHOS can bear tumor resistance for chemotherapy, radiotherapy, and targeted therapy, which also appears in NSCLC. Therefore, blocking this mitochondrial “compensation” may inhibit tumor resistance.

In a radiotherapy-resistant NSCLC model, FOXO3a is upregulated with the concomitant activation of PINK1/Parkin-mediated protective mitophagy; Inhibition of FOXO3a downregulates this mitophagy pathway and decreases radiotherapy tolerance in tumor cells, suggesting that the FOXO3a-PINK1/Parkin axis directly determines tumor response to radiotherapy through mitophagy levels regulation (Zhang et al., 2024). When FOXO3a is inhibited, the PINK1/Parkin-mediated protective mitophagy is also inhibited, showing that the tumor cells have a stronger response to radiotherapy. Under hypoxic conditions, HIF-1α induces SIRT1 expression, and sirtuin-1 is a deacetylase of HIF-1α and also of PGC-1α and the transcription factor PPAR-γ, which modulate mitochondrial biogenesis and mitochondrial function and ultimately form the SIRT1/PGC-1α/PPAR-γ axis that appears to be involved in hypoxia-triggered chemotherapy resistance by mediating metabolic rewiring and immune modulation (Xu et al., 2021).

### 3.3 Regulation of mitochondrial function by oncogenes (MYC, RAS) and tumor suppressors (p53, LKB1)

Among oncogenes, MYC and RAS are the central amplifying regulatory switches that lead to reprogrammed mitochondrial metabolism to drive malignant tumor proliferation. MYC- as a key transcription factor-is a central upstream regulator of both mitochondrial biogenesis and anabolic metabolism. Indeed, MYC directly binds to the promoter regions of many mitochondrial functional genes, including mitochondrial transcription factor A (TFAM), mitochondrial ribosomal protein genes, and genes that encode key rate-limiting enzymes responsible for glycolysis and glutaminolysis to activate mitochondrial biogenesis comprehensively.

MYC enhances the expression of glycolytic enzymes, such as hexokinase 2 (HK2) and lactate dehydrogenase A (LDHA), rapidly fueling aerobic glycolysis, thus dramatically increasing ATP production and providing various metabolic intermediates. Second, MYC also upregulates glutamine transporters and key enzymes of glutaminolysis; mitochondrial glutamine metabolism replenishes TCA cycle substrates to compensate for the defect induced by excessive glycolysis as demonstrated to ensure sufficient carbon and nitrogen supply to support nucleotide and lipid biosynthesis and cell proliferation. On the other hand, the other classic oncogenic switch, mutant RAS, cooperates with MYC in reshaping mitochondrial function. The activated RAS pathway stabilizes the MYC protein and amplifies its transcriptional regulatory effects and modulates mitochondrial membrane potential and oxidative phosphorylation (OXPHOS) activity. The RAS pathway is expected to modestly inhibit excess mitochondrial OXPHOS, reduce the accumulation of reactive oxygen species (ROS) to prevent oxidative stress-induced apoptosis, and maintain a low level of mitochondrial respiration to secure the cell’s basic energy supply. In other words, this secures the highly proliferating metabolic phenotype in cancer cells, thus facilitating tumor invasion and metastasis (Jose et al., 2011).

In contrast, the tumour suppressors p53 and LKB1 act as negative regulatory switches for tumour mitochondrial metabolic reprogramming. Through their respective downstream signalling pathways, they can reverse the Warburg effect and maintain mitochondrial metabolic homeostasis, thereby inhibiting malignant metabolic reprogramming in tumours. The classic tumour suppressor p53 is a key upstream sensor of mitochondrial metabolic stress; wild-type p53 can suppress the expression of core glycolytic genes such as glucose transporter 1 (GLUT1) and HK2 at the transcriptional level, thereby blocking aerobic glycolysis. Concurrently, p53 upregulates the expression of mitochondrial respiratory chain complex subunits and TCA cycle enzymes, enhancing the efficiency of mitochondrial oxidative phosphorylation (OXPHOS) and restoring normal levels of cellular oxidative metabolism (Liu and Gu, 2022). Furthermore, p53 regulates mitochondrial membrane permeability and activates the mitochondrial apoptotic pathway, linking mitochondrial metabolic dysregulation to cell cycle arrest and apoptosis in tumour cells, thereby inhibiting the unlimited proliferation of tumours. Once p53 is mutated or inactivated in tumours, its metabolic regulatory function is completely lost, leading to abnormal activation of glycolysis and mitochondrial dysfunction, thereby accelerating malignant tumour progression (Figure 1). While MYC-driven mitochondrial biogenesis has been extensively characterized in LUAD, recent studies suggest that MYC amplification in SCLC (particularly the MYC-amplified subtype) drives a similar OXPHOS-high metabolic state that renders these tumors vulnerable to mitochondrial targeting (Chalishazar et al., 2019).

**FIGURE 1:**
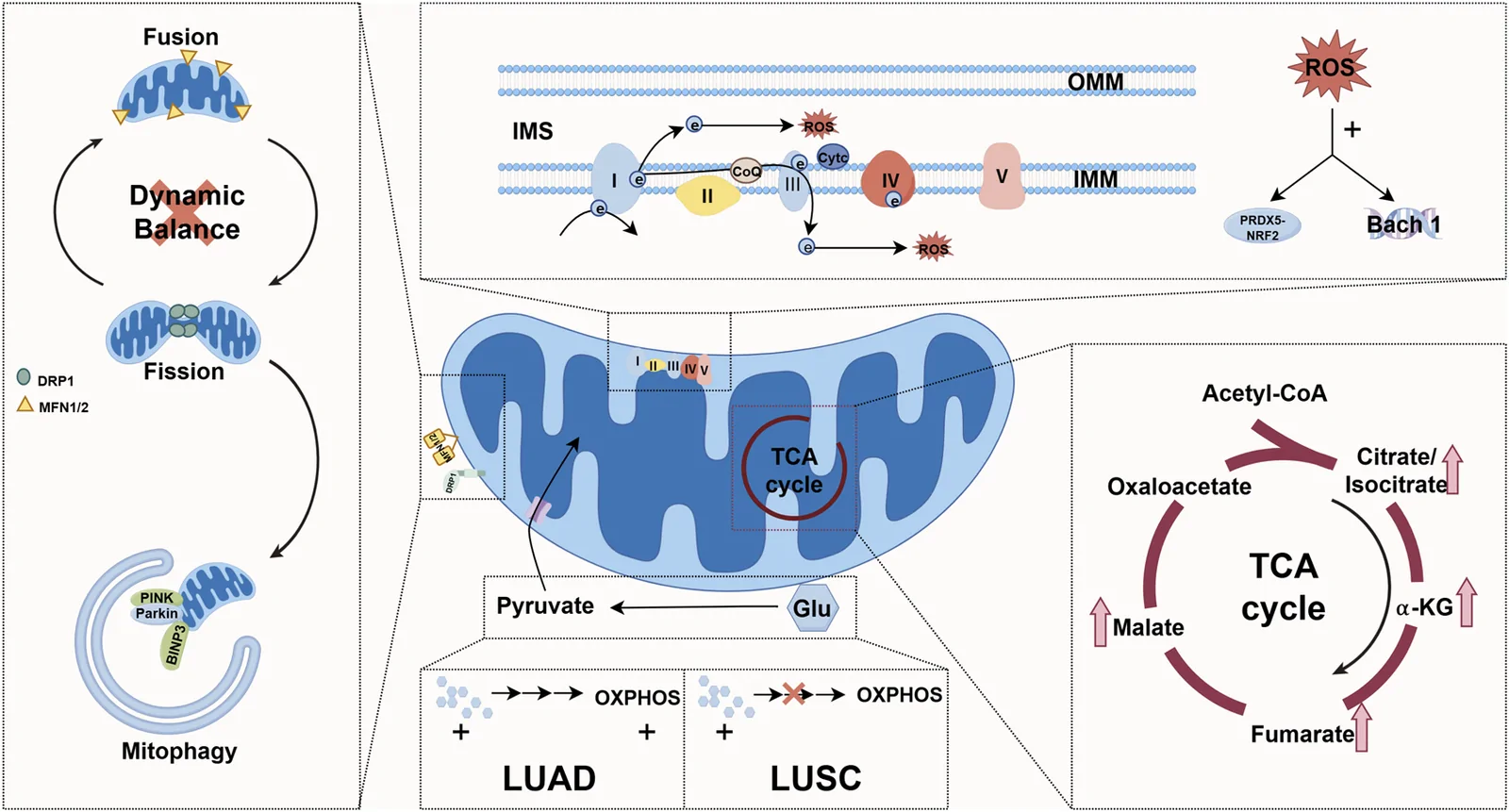
Illustrates the central metabolic-transcriptional axis driving adaptive drug resistance in lung cancer.

## 4 Mitochondrial metabolites as regulators of nuclear transcription

### 4.1 TCA cycle intermediates as cofactors of epigenetic enzymes (α-KG, succinate, fumarate)

#### 4.1.1 Competitive inhibition of epigenetic enzymes

As major enzymes of the TCA cycle, fumarate hydratase (FH) and succinate dehydrogenase (SDH) are mutated in certain human tumors, leading to the aberrantly elevated metabolic substrates fumarate and succinate, respectively. Fumarate and succinate were previously shown to compete with each other at α-KG-dependent dioxygenases and inhibit a wide range of essential enzymes, including histone demethylases and hydroxylases of the TET family, thus provoking genome-wide changes in histone and DNA methylation and, eventually, tumorigenesis (Xiao et al., 2025).

#### 4.1.2 Suppression of immune cell functions

Gain-of-function mutations in isocitrate dehydrogenase (IDH) in human tumors lead to producing the oncometabolite D-2-hydroxyglutarate (D-2HG), which drives tumorigenesis via epigenetic reprogramming. Mechanistically, tumor-derived D-2HG is taken up by CD8 (+) T cells and induces acute and reversible changes in their metabolism and anti-tumor functions. Other studies have identified the glycolytic enzyme lactate dehydrogenase (LDH) as a specific molecular target of D-2HG.

#### 4.1.3 Regulatory roles as signaling molecules

Mitochondrial TCA cycle intermediates are more than biosynthetic intermediates; they also act as signaling molecules to regulate chromatin modification, DNA methylation, hypoxic responses, and immune responses. Changes in their abundance can influence cell function and fate. Understanding metabolite-mediated signaling provides deeper insights into how metabolism impacts physiological processes and disease pathogenesis (martínez-Reyes and Chandel, 2025).

### 4.2 Acetyl-CoA production and histone acetylation

#### 4.2.1 Regulation of acetyl-CoA metabolism

Acetyl-CoA metabolism is finely tuned in space and time according to the cell’s nutrient and signaling needs. Mammalian cells regulate acetyl-CoA generation differently in the nucleus than in the cytoplasm. Previous reports have shown that post-translational modifications of acetyl-CoA-generating enzymes and their dynamic nuclear localization and recruitment to chromatin regulate acetyl-CoA supply for site-specific histone acetylation in specific gene regulation (Sivanand et al., 2025).

#### 4.2.2 p300/CBP-mediated drug resistance and stemness gene regulation

In malignant tumours such as lung cancer, acetyl-CoA is a rate-limiting substrate for the histone acetyltransferases p300/CBP. When mitochondrial metabolic flux is altered, leading to a substantial increase in acetyl-CoA production, p300/CBP can utilise this substrate to specifically elevate the levels of histone acetylation (e.g., H3K27ac) in the promoter regions of genes associated with the epithelial-mesenchymal transition (EMT) (Snail, Slug) and stemness genes (Sox2, Oct4, Nanog). This epigenetic modification relaxes chromatin structure, directly driving phenotypic switching in tumour cells and enhancing their resistance to chemotherapies such as cisplatin and to EGFR tyrosine kinase inhibitors (EGFR-TKIs).

#### 4.2.3 Glycolytic reprogramming regulates acetyl-CoA-dependent epigenetic modification

Metabolic reprogramming is a hallmark of activated T cells. Shifting cellular metabolism from oxidative phosphorylation toward aerobic glycol generates energy and metabolic intermediates for macromolecular biosynthesis in the service of clonal expansion and effector functions. Moreover, glycolytic reprogramming may control inflammatory gene expression via epigenetic remodeling. In LUSC, NOTCH pathway inactivation has been shown to rewire acetyl-CoA metabolism through downstream target genes, linking lineage plasticity to epigenetic reprogramming (Anusewicz et al., 2020).

### 4.3 NAD+/NADH ratio and sirtuin-mediated deacetylation

#### 4.3.1 SIRT3 mediates mitochondrial metabolism to regulate epigenetics

SIRT3 is the most abundant deacetylase in mitochondria, and its activity is critically dependent upon mitochondrially generated NAD+. SIRT3 inhibits the Warburg effect by deacetylation and inactivation of HIF-1α in lung cancer cells. Mitochondrial dysfunction or decreased NAD+ levels reduce SIRT3 activity, resulting in accumulation and nuclear translocation of HIF-1α, which stimulates transcription of glycolysis-related genes and induces metabolic resistance to chemotherapies (e.g., cisplatin) and EGFR-TKIs. Moreover, SIRT3 deacetylates and regulates mitochondrial metabolic enzymes (IDH2, SOD2), and is involved in the regulation of redox homeostasis and the protection of drug-resistant lung cancer cells from drug-induced oxidative stress (Zhao et al., 2025).

#### 4.3.2 NAD+/NADH ratio regulates tumor cell proliferation

Besides its essential function in ATP production, the electron transport chain (ETC) has recently attracted much attention due to its role in cell proliferation regulation. Among the four ETC complexes, complex I (CI)-mediated NAD+ regeneration is critical for cancer cell proliferation. CI-mediated NAD+ regeneration determines the intracellular NAD+/NADH ratio. A decreased ratio inhibits the activities of SIRT3 and SIRT7, preventing the expression of p21Cip1 at transcriptional and translational levels, and also drives tumor cells into a slowly proliferative ‘dormant’ state to escape from cytotoxic killing, an important escape mechanism for acquired resistance in lung cancer (Higurashi et al., 2025).

#### 4.3.3 NNMT-mediated NAD+ depletion and tumor phenotypic switching

The enzyme nicotinamide N-methyltransferase (NNMT) is frequently found to be significantly overexpressed in highly invasive lung cancer cells. NNMT indirectly decreases intracellular NAD+ levels by decreasing NAD+ precursors and subsequently inhibits the NAD+-dependent SIRT1/SIRT3 signaling pathway. This metabolic shift leads to enhanced acetylation (and modulation of activity) of tumor suppressors such as p53, and co-activates EMT-associated transcriptional programs, ultimately increasing drug resistance and distant migration of lung cancer cells (Kraus et al., 2014).

### 4.4 SAM availability and methylation reactions

#### 4.4.1 Dysregulated methionine cycle

The essential amino acid methionine plays a central role in cellular one-carbon metabolism and promotes the production of SAM. SAM is the major methyl donor in DNA methylation and plays a direct role in the epigenetic regulation of nuclear gene expression. Studies demonstrate that in lung cancer, during targeted resistance, metabolic flux changes in the methionine cycle lead to SAM supply fluctuations. Low SAM levels or poor use leads to DNA hypomethylation at specific tumor suppressor genes promoters to activate pro-survival factors or bypass activation genes (e.g., MET, AXL) and reduce the sensitivity of tumor cells to EGFR-TKIs (Lin et al., 2025).

#### 4.4.2 NNMT-mediated “methyl pool sequestration” and lung cancer EMT

NNMT uses SAM as a methyl donor to modify nicotinamide, participating in NAD+ metabolism regulation. Meanwhile, SAM provides methyl groups for histone methylation, directly contributing to epigenetic control of nuclear transcription. In lung cancer cells, NNMT-mediated SAM depletion alters histone H3K4 or H3K27 methylation, relieving transcriptional repression of mesenchymal genes such as Snail and Slug. Such metabolism-driven histone demethylation markedly enhances invasiveness and endows cancer cells with stem-like properties, enabling survival under therapeutic pressure.

### 4.5 ROS as signaling molecules activating transcription factors (NF-κB, NRF2, HIF-1α)

#### 4.5.1 ROS-mediated oxidation-O-GlcNAcylation cascade regulates ferroptosis

ROS play a key role in lipid peroxidation and the initiation of ferroptosis, and can significantly influence tumour resistance to chemotherapy. Previous studies have confirmed that O-linked N-acetylglucosamine transferase (OGT), the key enzyme mediating protein O-GlcNAcylation, acts as an important sensor of ROS during ferroptosis. ROS can induce oxidation of residue C845 in the catalytic domain of OGT, thereby activating OGT. Activated OGT catalyses the O-GlcNAcylation of FOXK2, enhancing its interaction with importin α, promoting the nuclear translocation of FOXK2 and its binding to the SLC7A11 promoter region. This cascade ultimately upregulates the transcriptional level of SLC7A11, thereby inhibiting ferroptosis.

#### 4.5.2 ROS detoxification contributes to targeted therapy resistance in lung cancer

The use of EGFR-TKIs has brought significant survival benefits to patients with advanced EGFR-mutant non-small cell lung cancer (NSCLC). However, tumour cells inevitably develop acquired resistance, which severely limits the clinical application of this class of drugs. Experimental studies have shown that prolonged exposure of lung cancer cells to EGFR-TKIs can induce a drug-resistant phenotype, characterised by abnormal cholesterol accumulation within lipid rafts. This abnormal accumulation of cholesterol further promotes the EGFR–Src interaction, mediating the reactivation of the EGFR/Src/Erk signalling pathway, which in turn regulates SP1 nuclear translocation and ERRα re-expression. Subsequent studies have confirmed that ERRα is a direct target gene of SP1. At the functional level, re-expressed ERRα maintains the proliferative capacity of drug-resistant tumour cells by regulating ROS detoxification. The cholesterol-lowering agent lovastatin and the ERRα inverse agonist XCT790 can effectively reverse tumour cell resistance to gefitinib and osimertinib in both *in vitro* and *in vivo* models, providing a novel intervention strategy for drug resistance in targeted therapy for lung cancer (Pan et al., 2022).

#### 4.5.3 Synergy between ROS and glycation/carbonyl stress promotes tumorigenesis

Oxidative stress, carbonyl stress and glycation stress collectively constitute a cellular stress regulatory network, and the regulatory role of this network in tumour progression is attracting increasing attention. Oxidative stress can activate various nuclear transcription factors, such as NF-κB, HIF-1α and Nrf2, via ROS, triggering changes in the expression of hundreds of genes and inducing the malignant transformation of normal cells. Studies have shown that ROS-mediated lipid peroxidation can generate large quantities of dicarbonyl compounds, inducing glycation/carbonyl stress and causing carbonyl damage to biomacromolecules such as proteins, DNA and lipoproteins.

## 5 Role of transcriptional and epigenetic reprogramming in drug-resistant lung cancer

### 5.1 LUAD: transcriptional and epigenetic plasticity under therapeutic pressure

#### 5.1.1 Genetic and chromatin drivers of lineage plasticity

Some tumor cells, in response to long-term EGFR-TKI exposure, acquire a drug-tolerant persister (DTP) state, which is characterized by reduced proliferation, preserved original clonal structure, and reversible non-genetic resistance, with extensive transcriptional reprogramming that activates stress-adaptive pathways such as NF-κB and STAT3. This demonstrates that adaptive resistance is primarily caused by cellular plasticity rather than by irreversible genomic evolution.

Genetic changes in lineage-determining pathways further drive the phenotypic plasticity of LUAD. Experimental studies have shown that simultaneous deletion of Rb1 and Trp53 creates a cellular environment that drives lineage transition, and that an additional deletion of PTEN promotes the acquisition of diverse histological phenotypes, including SCLC-like tumors, and at the same time leads to substantial transcriptional heterogeneity within a single lesion (Zhang et al., 2022a). Chromatin remodelers also regulate lineage plasticity. As a core component of the SWI/SNF complex,the loss of ARID1A increases chromatin accessibility and reshapes enhancer landscapes in a Kras/Tp53-driven LUAD model, thereby triggering large-scale transcriptional reprogramming and activating glycolytic pathways (Liu et al., 2022). Similarly, SMARCA4 deficiency alters enhancer architecture and immune-related transcriptional programs, indicating that SWI/SNF-mediated chromatin regulation plays a crucial role in determining cellular identity and therapeutic response (Wang et al., 2025b).

#### 5.1.2 Neuroendocrine transcriptional reprogramming

Activation of neuroendocrine transcriptional program drives EGFR-mutant LUAD to SCLC phenotype. Transcription factors specific to the lineage, ASCL1 and NEUROD1, are key regulators of neuroendocrine differentiation and control a variety of cellular states in SCLC and related neuroendocrine tumors (Duplaquet et al., 2023).

More recent studies have shown that the conversion of neuroendocrine subtypes is not a static event but a highly dynamic one. Inactivation of KDM6A changes the ratio of H3K4me1 and H3K27me3 modifications in neuroendocrine regulatory regions and creates a chromatin environment that favors subtype conversion and enables interconversion of ASCL1-and NEUROD1-dominant transcriptional state (Duplaquet et al., 2023).

#### 5.1.3 Epigenetic remodeling during lineage transition

Epigenetic remodeling is a central mechanism linking therapeutic stress to lineage plasticity.

KDM6A-mediated regulation of enhancer activity is a classic example of epigenetic regulation. By modulating the levels of H3K27me3 and H3K4me1 modifications on neuroendocrine regulatory elements,KDM6A regulates the chromatin accessibility of lineage-specific enhancers and participates in maintaining or switching ASCL1-and NEUROD1-related transcriptional programs (Duplaquet et al., 2023).

“Beyond neuroendocrine differentiation, epithelial–mesenchymal transition (EMT) is another core adaptive reprogramming pathway. In EGFR-TKI-resistant LUAD cells, upregulation of DCLK1 induces EMT, manifested by downregulation of E-cadherin and upregulation of mesenchymal markers such as ZEB1, vimentin, and N-cadherin,ultimately enhancing the migratory and invasive capabilities of tumor cells (Yan et al., 2023). Similarly, in the process of acquiring osimertinib resistance in an EGFR-driven mouse LUAD model,large-scale transcriptional reprogramming occurs, accompanied by the enriched activation of the EMT pathway and Wnt/β-catenin signaling (Yan et al., 2023).

#### 5.1.4 Drug-tolerant intermediate states

In EGFR-mutant patient-derived xenograft models, long-term treatment with erlotinib can induce the formation of DTP tumours. These tumours retain their original genomic characteristics whilst exhibiting reversible drug-resistant phenotypes and undergoing extensive transcriptional remodelling. Previous studies based on the PC9 cell model have further confirmed that reversible histone modifications, together with an adaptive transcription factor network comprising NF-κB, TEAD, YAP and NRF2, may collectively contribute to the establishment and maintenance of the DTP state (Moghal et al., 2022).

Single-cell transcriptomic analysis has further confirmed that a DTP-like cell subpopulation, accounting for approximately 4 per cent, is already present in untreated tumours. These cells exhibit intermediate transcriptional characteristics between initial tumour cells and mature DTP cells, suggesting that therapeutic stress primarily enriches pre-existing plastic cellular states within the tumour, rather than inducing entirely new drug-resistant cell subpopulations from scratch (Moghal et al., 2022).

Cancer stem cell (CSC)-like subpopulations also drive adaptive drug resistance. In EGFR-TKI-resistant LUAD, low Shisa3 expression is closely associated with enhanced tumor stemness, increased spheroid formation capacity, and resistance to targeted therapy. Conversely, restoring Shisa3 expression can inhibit tumor self-renewal, migration, and invasion by suppressing the FGFR/AKT/mTOR signaling axis, thereby reversing the resistance phenotype.

### 5.2 LUSC: squamous transdifferentiation and therapy resistance

Chromatin remodeling is among the earliest events in squamous transdifferentiation, and LKB1 loss is a strong driver of AST. Indeed, the Kras; Lkb1 mouse model demonstrates that LUAD can undergo *bona fide* LUAD-to-LUSC lineage reprogramming through the intermediate state of mAd-SCC rather than just a histological illusion (Han et al., 2014). Loss of LKB1 induces redox imbalance via the ROS pathway, driving metabolic stress-induced squamous transdifferentiation and linking metabolic adaptation, lineage plasticity and treatment resistance (Li et al., 2015). Depletion of SMARCA4, a core subunit of the SWI/SNF complex, alters the enhancer landscape genome-wide and decreases global chromatin accessibility. Alternatively, downregulation of inflammation and immunity genes results in a “immunologically cold microenvironment”, which confers resistance to immune checkpoint therapy (Wang et al., 2025b).

Squamous transdifferentiation in EGFR-mutant LUAD does not depend on *de novo* secondary driver mutations; rather, it centres on the reprogramming of the PI3K/AKT, MYC and PRC2 transcriptional networks. Persistently activated PI3K/AKT and MYC act synergistically with EZH1/EZH2 to stabilise squamous-specific transcriptional programmes, directly inducing resistance to osimertinib; inhibition of either the EZH1/2 or PI3K/AKT pathways can block transdifferentiation and restore sensitivity to EGFR inhibitors, whilst SETDB1-mediated AKT methylation further amplifies this resistance circuit (Guo et al., 2019).

Squamous transdifferentiation preserves lineage memory; LUSCs formed via transdifferentiation inherit a large proportion of the methylation and transcriptional characteristics of the original LUAD. The vast majority of resistant squamous cell carcinomas arise from the reprogramming of pre-existing tumour clones, rather than the replacement by entirely new clones. LKB1-deficient tumors undergo transdifferentiation while upregulating molecules from the DNMT and TET families, leading to altered levels of 5-methylcytosine and 5-hydroxymethylcytosine modifications; blockade of the STAT3–CXCL5 axis suppresses lineage conversion (Xue et al., 2024); SMARCA4 mutations cause altered chromatin accessibility at >20,000 genomic loci, mainly affecting NF-κB-related inflammatory enhancers, resulting in tumor immune evasion and resistance to immunotherapy (Wang et al., 2025b).

LKB1-deficient NSCLC is characterized by DNA repair defects and PARP1 hyperactivation. PARP1 inhibitors elicit an antitumor effect through synthetic lethality, while restoring IFN-γ–STAT1–mediated antitumor immunity (Long et al., 2022). The PI3K/AKT pathway induces the expression of ABC transporters via an NRF2-dependent mechanism, which may be responsible for the multidrug-resistant phenotype of transdifferentiating tumors.

### 5.3 SCLC: neuroendocrine state switching and epigenetic adaptation

SCLC exhibits extreme cellular plasticity and is highly prone to rapidly developing treatment resistance; despite its relatively simple genome, it displays extensive transcriptional and epigenetic heterogeneity. Dynamic switching between neuroendocrine states and epigenetic reprogramming jointly regulate tumour progression, metastasis and resistance, and are synergistically controlled by driver mutations, lineage-specific transcription factors and chromatin remodelling proteins (Ghafoor et al., 2025; Duplaquet et al., 2023).

Virtually all SCLC cases harbour dual deletions of TP53 and RB1, which disrupt cell cycle and genomic stability, providing the genetic basis for lineage plasticity (Duplaquet et al., 2023). Functional loss mutations in KMT2C occur frequently in advanced SCLC; this molecule regulates DNMT3A transcription via H3K4 methylation. Inactivation of KMT2C downregulates DNMT3A, lifting the methylative repression of metastasis-associated genes such as MEIS and HOX; supplementation with the methyl donor SAM or restoration of DNMT3A can reverse the highly metastatic tumour phenotype. NFIB amplification upregulates genome-wide chromatin accessibility, activates transcriptional programmes associated with neural differentiation and metastasis, and drives extensive epigenetic remodelling (Na et al., 2022). The loss of TP53/RB1, combined with abnormalities in KMT2C, DNMT3A and NFIB, collectively contributes to the highly plastic transcriptional profile observed in advanced SCLC (Duplaquet et al., 2023).

Based on lineage-specific transcription factors, SCLC is classified into four categories: SCLC-A, SCLC-N, SCLC-P and inflammatory SCLC-I, of which SCLC-A and SCLC-N belong to the high neuroendocrine subtypes, whilst SCLC-P and SCLC-I are low neuroendocrine subtypes; These subtypes do not represent fixed cell types, but rather dynamic and variable transcriptional states; subtype heterogeneity directly accounts for differences in treatment response (Ghafoor et al., 2025).

MYC activation is the key driver of the transition from SCLC-A to the highly aggressive SCLC-N; following this transition, tumour differentiation decreases and clinical prognosis worsens (Duplaquet et al., 2023; Voigt et al., 2021); KDM6A loss disrupts the balance of H3K27me3/H3K4me1 modifications, creating a chromatin microenvironment conducive to subtype switching and enhancing the tumour’s resistance to drugs (Duplaquet et al., 2023). Metastatic SCLC is characterised by widespread low methylation of genomic DNA and histones; KMT2C defects trigger extensive epigenetic dysregulation, leading to the sustained activation of genes associated with development and metastasis; NFIB-mediated chromatin opening is a key prerequisite for the acquisition of metastatic capacity by the tumour (Yang et al., 2018; Na et al., 2022). HDAC and LSD epigenetic inhibitors can reshape the differentiation state of SCLC cells, upregulate antigen-presenting molecules, and significantly enhance the efficacy of PD-1 immune checkpoint blockad (Hiatt et al., 2022) (Figure 2). Overall, TP53/RB1 loss constitutes the genetic framework of SCLC, whilst disruption of the KMT2C-DNMT3A pathway and NFIB amplification drive global epigenetic re-programming, mediating subtype switching, metastasis and immune evasion; targeting these epigenetic pathways in combination with radiotherapy and chemotherapy can reverse drug resistance (Ghafoor et al., 2025; Hiatt et al., 2022; Duplaquet et al., 2023; Na et al., 2022).

**FIGURE 2:**
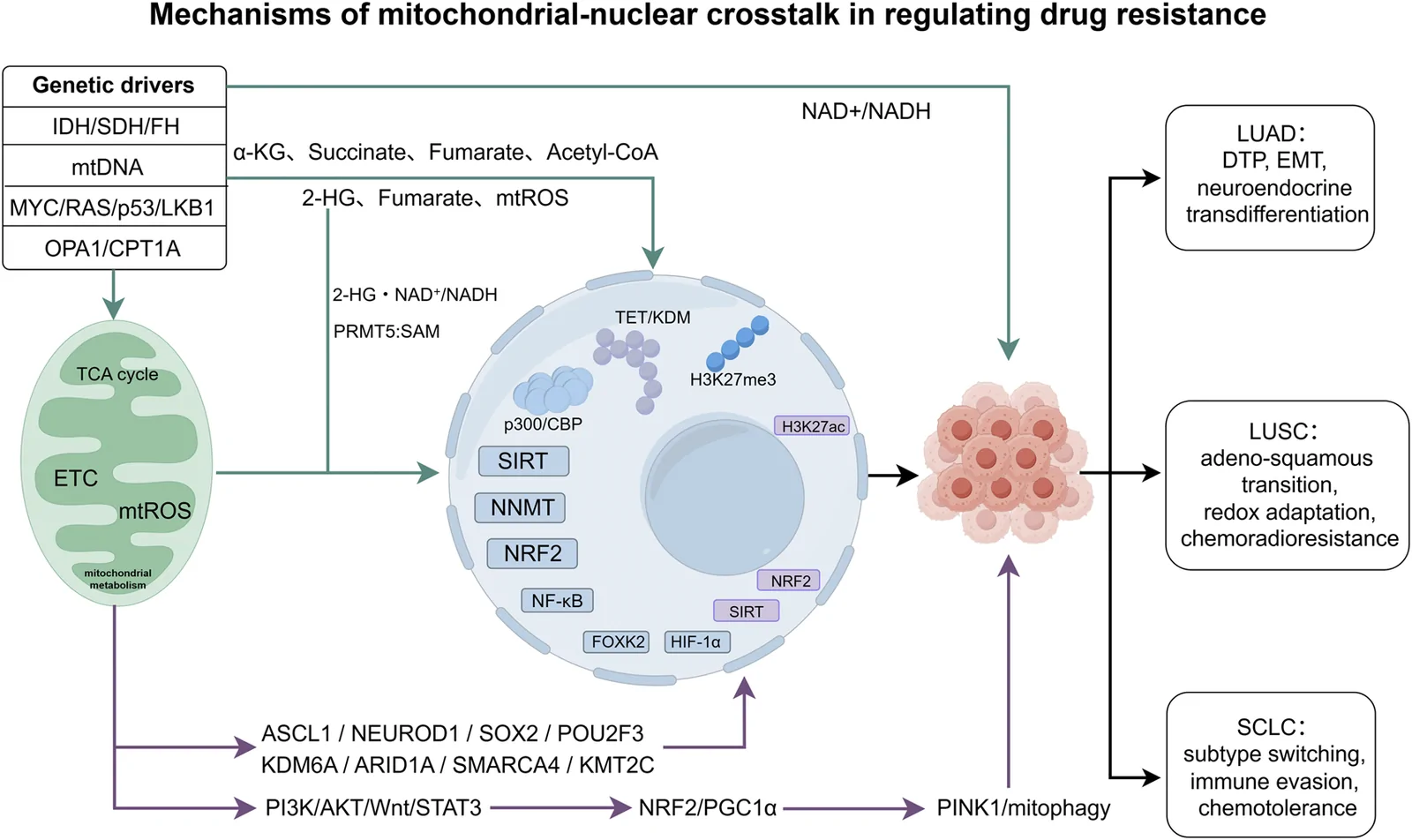
Visually summarizes subtype-specific mitochondrial reprogramming patterns underlying therapeutic plasticity.

## 6 Therapeutic strategies targeting mitochondrial metabolism to overcome resistance

### 6.1 Targeting mitochondrial metabolic vulnerabilities

The core driver of EGFR-TKI resistance in EGFR-mutant LUAD is mtOXPHOS upregulation; the SIRT1-dependent mitochondrial oxidative phosphorylation pathway supports the survival of drug-tolerant CSCs cells. SIRT1 inhibition by tenovin-6 and mitochondrial translation blockage by tigecycline disrupted this metabolic dependency, restored gefitinib sensitivity, and achieved sustained tumor regression in a xenograft model. Moreover, the AKR1B1–STAT3–SLC7A11 antioxidant pathway and ACLY-mediated citrate-acetyl-CoA metabolic axis are promising targets to reverse and delay resistance to targeted therapy (Phutela et al., 2025; Zhang et al., 2021) (Figure 3).

**FIGURE 3:**
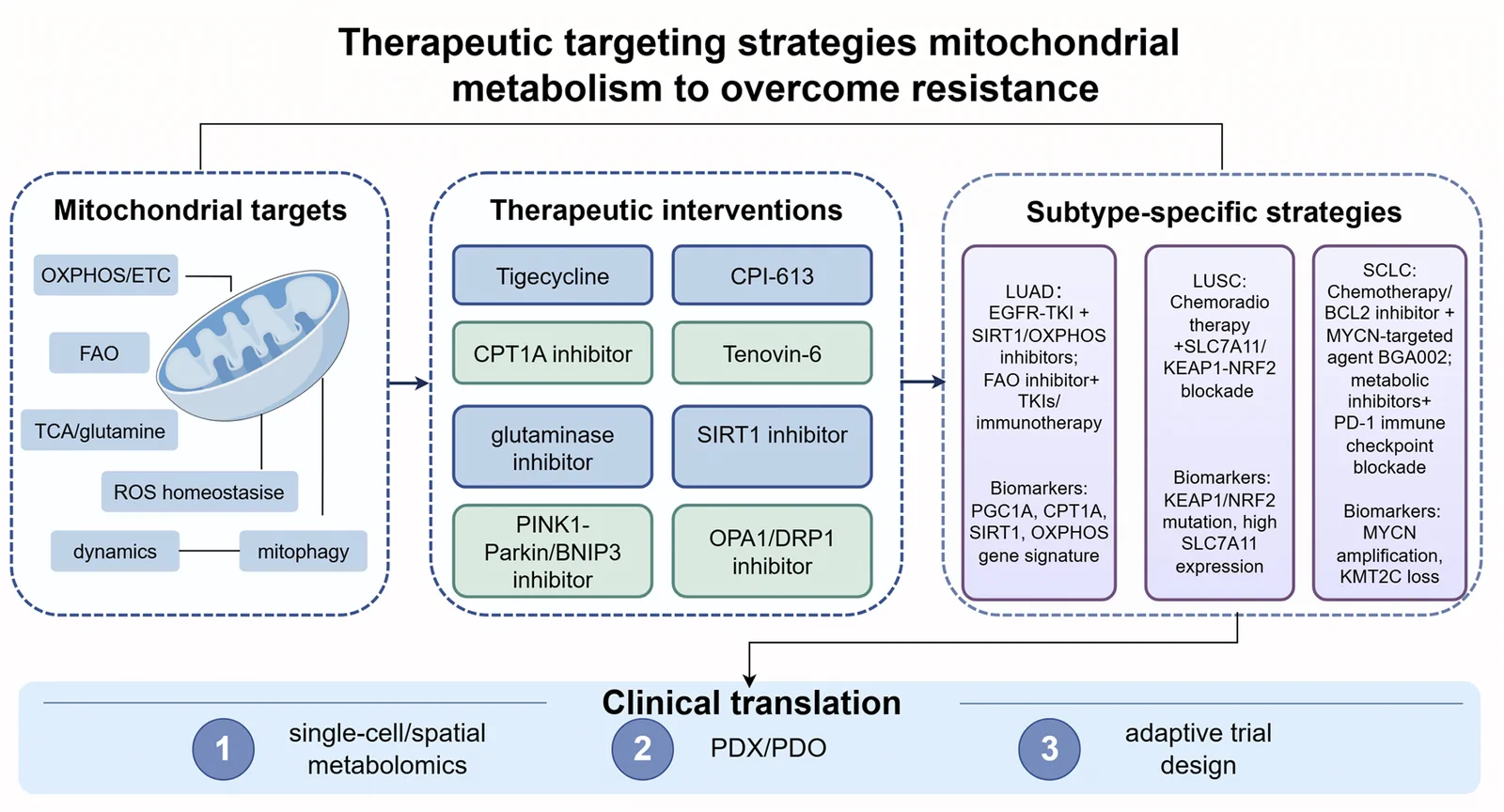
Therapeutic targeting mitochondrial metabolism to overcome resistance.

Besides OXPHOS addiction, FAO confers metabolic plasticity to LUAD and mediates TKI resistance. CPT1A, the rate-limiting enzyme of FAO, activates the c–Myc–NRF2–GPX4 pathway and thus maintains tumor stemness and resistance to ferroptosis. Genetic or pharmacological inhibition of CPT1A increases lipid peroxidation in cells, inducing ferroptosis in resistant cells and thus providing a potential combinational therapeutic strategy.

The mechanisms of LUS resistance primarily involve redox reprogramming. Indeed, inactivating mutations of KEAP1 lead to constitutive activation of NRF2, which transcriptionally upregulates SLC7A11 to enhance cysteine uptake and glutathione synthesis, thereby reducing oxidative stress and ultimately resulting in resistance to radiotherapy and chemotherapy. This protective antioxidant effect can be reversed by sulfasalazine through the inhibition of the X-transporter (Jeong et al., 2016).

Mitochondria-targeted research frameworks for SCLC are not well established; activation of the MYC family is a crucial driver of disease progression and resistance to platinum-based etoposide, and MYCN overexpression mediates multidrug resistance. Targeted BGA002 drugs inhibit oncogenic signaling and reverse resistance (Grunblatt et al., 2020). ETC-targeted regimens have not yet been systematically validated (Table 2). A variety of experimental mitochondrial-targeted therapeutic candidates under pre-clinical and early-phase clinical development are summarized in (Table 3).

**TABLE 2:** Comparative analysis of targeted metabolic strategies across three lung cancer subtypes.

Comparison dimension	Lung adenocarcinoma (LUAD)	Lung squamous cell carcinoma (LUSC)	Small cell lung cancer (SCLC)
Differences in Targeted Metabolic Strategies in Clinical Trials	EGFR-TKI Combined with Metabolic Intervention: Targeting DPP4 inhibitors or MEK inhibitors to block NRF2-mediated resistance (ZHANG et al., 2025b). ERRα Inverse Agonists: Using XCT790 combined with lovastatin to overcome EGFR-TKI (gefitinib/osimertinib) resistance (Pan et al.). Serine Biosynthesis Inhibitors: Blocking serine synthesis to inhibit tumor growth, targeting metabolic compensation caused by POLG mutations (Cararo Lopes et al., 2025). IDH Inhibitors: Targeting IDH1/2 mutations to reduce 2-HG levels and reverse epigenetic blockade (Zhang et al., 2023)	Mitochondrial Fusion Inhibitors: Targeting the p32/OPA1 axis to induce mitochondrial fission and restore cisplatin sensitivity (Yu et al., 2024). Mitophagy Inhibitors: Combining FOXO3a or PINK1/Parkin inhibitors to overcome radiotherapy and chemotherapy resistance (Zhang et al., 2024). ROS Modulators: Utilizing PRDX5-NRF2 or Bach1 pathways to disrupt adaptive oxidative stress balance (Qian et al., 2022; Hao et al., 2021). SIRT1 Inhibitors: Blocking the SIRT1/PGC-1α/PAR-γ axis to inhibit hypoxia-induced metabolic adaptation (Xu et al., 2021)	OXPHOS Inhibitors: Using oligomycin and other inhibitors targeting the high OXPHOS dependence of CSC subpopulations (Gao et al., 2016). mtDNA-Targeted Platinum Agents: Using platinum-mtDNA conjugates to enhance mitochondrial apoptotic signaling and reverse cisplatin resistance (van Gisbergen et al., 2015). ABC Transporter Inhibitors: Targeting ABCB1/ABCG2 overexpression to restore intracellular drug concentration (Robey et al., 2018). Mitophagy Blockade: Combining RFX2-BNIP3 or PINK1/Parkin inhibitors to eliminate resistant subpopulations (Cao et al., 2025)

**TABLE 3:** Experimental agents targeting mitochondrial metabolism in clinical development.

Therapeutic agent/Platform	Developmental stage	Mechanism of action (MoA)	Target population and key characteristics	Ref
LND-SS-Pt-TPP/HA-CD nanopro-drug	Preclinical	GSH-responsive co-release of LND and Pt (IV) via TPP-mediated mitochondrial localization; synergistically disrupts OXPHOS and glycolysis to overcome drug resistance	Cisplatin-resistant NSCLC (A549/DDP); tumors with high CD44 expression	(Lu et al., 2024)
Mito-Lonidamine (Mito-LND)	Preclinical	TPP-modified LND enhances mitochondrial accumulation; inhibits OXPHOS, induces autophagy, and promotes ROS generation to suppress primary tumors and metastasis	NSCLC and brain metastasis models; no apparent toxicity	(Zhang et al., 2022b)
Mito-Honokiol (Mito-HNK)	Preclinical	Targets Complex I and inhibits STAT3 phosphorylation; blocks OXPHOS signaling; exerts synergistic effects when combined with Mito-LND.	Highly aggressive NSCLC and brain metastasis; no apparent toxicity; potential application for chemoprevention	(Zhang et al., 2022b; Pan et al., 2018)
TPH/PTX nanomicelles	Preclinical	HA-mediated CD44 targeting and TPP-guided mitochondrial localization; delivers paclitaxel to mitochondria to induce apoptosis and overcome multidrug resistance (MDR)	MDR NSCLC (A549/ADR) and breast cancer lung metastasis models	(Wang et al., 2021)
Monofunctional Pt complexes (e.g., OPT)	Preclinical	TPP modification enhances mitochondrial accumulation; exerts anti-tumor effects via energy metabolism disruption and cytochrome c release	NSCLC (A549) and *in vivo* models; effective against both cisplatin-sensitive and -resistant phenotypes with reduced toxicity	(Zhu et al., 2019)
Mito-BEZ (Bezafibrate derivative)	Preclinical	Activates the PGC-1α/PPAR complex to regulate lipid metabolism; suppresses tumor OXPHOS and bolsters CD8^+^ T cell function (dual metabolic-immune strike)	LUAD models; effectively suppresses primary tumors and metastasis; immunomodulatory with no apparent toxicity	(Pan et al., 2025)
KHS101 (HSPD1 inhibitor)	Preclinical	Targets HSPD1 to trigger OXPHOS collapse and halt proliferation; highly dependent on SLC6A8/COX5B expression	NSCLC; highly sensitive in OXPHOS-dependent tumors; SLC6A8/COX5B serve as potential biomarkers for patient stratification	(Parma et al., 2021)
IACS-010759 ± Trametinib	Preclinical and Clinical	Blocks OXPHOS to overcome MEK inhibitor resistance in KRAS-mutant NSCLC; combined therapy significantly reduces tumor burden and extends survival	KRAS-mutant, high OXPHOS-dependent NSCLC resistant to MEK monotherapy	(He et al., 2023) (Feng et al., 2023)
Mitomet	Preclinical	Mitochondria-targeted metformin; amplifies ROS generation to selectively eradicate LKB1-deficient cells (1,000-fold higher potency than standard metformin)	Highly aggressive LKB1-deficient NSCLC subtypes; no apparent toxicity	(Shameem et al., 2023)
Demethylzeylasteral (T-96)	Preclinical	Targets LRPPRC to block OXPHOS complex biosynthesis, depleting ATP production and inducing apoptosis	LUAD models; potential precision efficacy in patients with high LRPPRC expression	(Wang et al., 2025a)
Sculponeatin A (sptA)	Preclinical	Induces targeted degradation of mitoSTAT3, disrupting mitochondrial function and triggering apoptosis; highly dependent on WWP2	NSCLC models; requires careful dosing due to potential cardiotoxicity risk (narrow therapeutic window)	(Wan et al., 2025)
CCCh-560-TPP	IND-enabling/Phase I prep	TPP-modified pyrrole-imidazole polyamide binds mtDNA ATP6 mutations; exerts broad-spectrum anti-tumor effects via ROS overload and apoptosis induction	Advanced metastatic pan-cancers (including NSCLC) driven by mtDNA mutations; highly effective in high-ROS microenvironments	(Nagase, 2025)

### 6.2 Targeting mitochondrial dynamics and mitophagy

Small-molecule mitochondrial-targeted drugs (Mito-LND, Mito-HNK) can induce ROS accumulation and autophagic cell death, thereby inhibiting the proliferation and metastasis of LUAD (Cheng et al., 2019). Targeting mitochondrial translation or SIRT1 can efficiently eliminate mtOXPHOS-dependent CSCs and reverse EGFR-TKI resistance (Sun et al., 2019), The clinical translational value of key regulators of mitochondrial dynamics (DRP1, OPA1, PINK1/Parkin) remains to be validated.

In LUSC, mitochondrial OXPHOS inhibitors inhibit the cellular energy supply and induce autophagic cell death to retard the tumor progression. Dual blockade of OXPHOS and FAO also shows broad antitumor activity (Cheng et al., 2019).

### 6.3 Targeting TCA cycle-associated metabolism

Metabolites of TCA cycle mediate the cross-talk between mitochondrial metabolism and epigenetic regulation in LUAD. The key intermediate α-ketoglutarate (α-KG) regulates mitochondrial bioenergetics and chromatin modification and has gender-dependent regulatory effects on tumor growth and immune microenvironment in KRAS-driven LUAD (Alcaraz et al., 2025). Notably, EGFR-TKI resistance is primarily attributed to abnormal regulation of the KEAP1/NRF2 and antioxidant pathways; to date, there is no direct evidence confirming that TCA cycle remodeling or IDH mutations are involved in this resistance process (Zhang et al., 2021).

### 6.4 Combination therapy

Targeting mitochondrial metabolism can produce synergistic effects with conventional targeted therapy to overcome drug resistance in LUAD. EGFR-TKIs in combination with SIRT1 inhibitors or mitochondrial translation inhibitors can eliminate drug-resistant CSC subpopulations (Sun et al., 2019). Osimertinib in combination with OXPHOS inhibitors is a promising preclinical therapeutic strategy, but there is no clinical evidence to support this at present (De Rosa et al., 2015). CPT1A inhibition also promotes ferroptosis in drug-resistant LUAD, providing a mechanistic basis for the combination of FAO inhibitors and TKIs.

The most well-established mitochondrial-related combination therapy strategy in the medical field of LUSC is the combination of radio/chemotherapy with SLC7A11 pathway blockade to effectively reverse the treatment resistance mediated by antioxidant mechanisms for NRF2-mutant LUSC (Zhang et al., 2021).

Most metabolic combination therapy regimens for SCLC are still in the preclinical research stage. The BCL2 inhibitor-chemotherapy combination has demonstrated some antitumor efficacy but has not yet been clinically validated and lacks corresponding predictive biomarkers.

### 6.5 Translational barriers

However, the clinical translation of mitochondrial targeting strategies faces significant obstacles, which must be addressed alongside their preclinical prospects. To date, no mitochondrial-targeting drugs have completed phase II/III clinical trials in the field of lung cancer, and most of the evidence cited in this review comes from cell line and xenograft models; these data should be interpreted with caution and not extrapolated to clinical efficacy without critical evaluation. Currently, the following four major challenges limit their clinical advancement: (1)Narrow therapeutic window—Systemic OXPHOS inhibition or ROS modulation exhibits inherent toxicity towards normal tissues with high energy demand (cardiomyocytes, neurons, hematopoietic stem cells),thereby limiting the dosage and duration of monotherapy. (2) Compensatory metabolic remodeling—Inhibition of a single pathway often triggers compensatory bypasses (e.g., upregulation of glycolysis after inhibition of OXPHOS), highlighting the necessity of rational combination drug therapy rather than metabolic monotherapy. (3) Lack of Biomarkers—Metabolic dependency lacks easily detectable biomarkers to identify beneficiary populations. Nevertheless, emerging genomic and metabolic surrogate markers provide promising directions for patient stratification: KEAP1/LKB1 mutation status can be used to identify antioxidant-dependent LUSC subtypes that are sensitive to ROS regulatory strategies; MYC amplification characterizes a subgroup of SCLC with high OXPHOS expression, which may preferentially respond to mitochondrial respiratory inhibitors; and DTP-related OXPHOS characteristics in EGFR-mutant LUAD help identify tumors that may benefit from combined metabolic blockade. Complementary technologies, including metabolomics analysis and hyperpolarized ^13^C-pyruvate PET imaging, need further validation of their value as predictive tools. (4) Drug Resistance Recurrence—Metabolic intervention may only delay, rather than prevent, the occurrence of secondary genetic resistance, suggesting that its optimal application strategy should be as an adjuvant combination therapy partner during the reversible DTP window period, rather than as an independent salvage treatment.

## 7 Conclusion

Classical resistance models centred on single driver gene mutations cannot fully explain the dynamic evolutionary characteristics of drug resistance across all lung cancer subtypes; this has made non-genomic adaptive mechanisms a key research focus in the field of tumour drug resistance in recent years. This review innovatively constructs a theoretical framework centred on the metabolic-transcriptional axis: therapeutic stress can induce extensive mitochondrial metabolic reprogramming in tumour cells, which in turn reshapes intracellular nuclear epigenetic and transcriptional profiles, endowing tumours with long-term adaptive plasticity and ultimately leading to the formation of stable yet modifiable drug-resistant phenotypes.

Several key targets—mitochondrial oxidative phosphorylation (OXPHOS), the TCA cycle, glutamine metabolism, and mitochondrial dynamics—have yielded significant synergistic anti-resistance effects with chemotherapy, targeted therapy, and immunotherapy. However, many technical and mechanistic bottlenecks limit clinical translation of these combined intervention strategies. Tumors keep remodeling their metabolic-transcriptional regulatory network under continuous drug stimulation, so isolated inhibition of any single metabolic pathway does not provide a substantial survival advantage for tumors, making complete reversal of drug resistance difficult.

Therefore, developing future therapeutics requires a shift in focus from the classical research paradigm on single isolated metabolic nodes toward multidimensional combined intervention strategies at the level of the entire adaptive network. This will inherently prevent the development and maintenance of persistent drug-resistant cell populations, opening up a new avenue for precise intervention against drug resistance in lung cancer.

With rapid development in single-cell sequencing, spatial omics, metabolomics, transcriptomics, and multi-omics integrative analysis, the systemic and precise characterization of metabolic dependencies and transcriptional adaptive states across different lung cancer patients and resistance stages has become increasingly possible. More importantly, increasingly compelling evidence indicates that tumor resistance is not a consequence of a few isolated genetic events but a dynamic process involving continuous remodeling of metabolic states, transcriptional programs, and epigenetic regulatory networks under therapeutic stress. Thus, static driver-mutation-based therapeutic paradigms are insufficient to explain resistance emergence and evolution. The integration of genomic, metabolomic, transcriptomic, and epigenomic information will allow future precision medicine to construct patient-specific “metabolic–transcriptional atlases” that dynamically represent the evolution of adaptive states across treatment stages. This would allow better patient stratification, real-time monitoring of therapeutic responses, and accurate prediction of resistance risk, thereby giving a more robust theoretical basis for individualized combination therapies.

Indeed, the core paradigm of lung cancer treatment is shifting from merely inhibiting driver mutations to systematically and rigorously characterizing dynamic metabolic–transcriptional adaptive networks under therapeutic stress and intervening in their evolutionary trajectories. Therefore, therapeutic targets should expand beyond tumor cells to include the metabolic–transcriptional resilience and adaptive plasticity acquired during treatment. This brings up a natural new challenge: dynamically modulating metabolic–transcriptional adaptation in tumor cells and directly interrupting their ability to adapt continuously under therapeutic stress, which may be one of the most significant breakthroughs in overcoming adaptive resistance and improving long-term lung cancer patient outcomes.
